# Affective prosody and facial emotion recognition in first-episode schizophrenia: Associations with functioning & symptoms

**DOI:** 10.1016/j.scog.2019.100153

**Published:** 2019-05-25

**Authors:** Kelsey A. Bonfils, Joseph Ventura, Kenneth L. Subotnik, Keith H. Nuechterlein

**Affiliations:** a VISN 4 Mental Illness Research, Education, & Clinical Center (MIRECC), VA Pittsburgh Healthcare System, 4100 Allequippa St., Pittsburgh, PA, United States; b University of Pittsburgh, Department of Psychiatry, 3811 O'Hara Street, Pittsburgh, PA, United States; c Aftercare Research Program, Jane and Terry Semel Institute for Neuroscience and Human Behavior at UCLA, UCLA Department of Psychiatry and Biobehavioral Sciences, 300 UCLA Medical Plaza, Los Angeles, CA, United States; d Department of Psychology, UCLA, Los Angeles, CA, United States

**Keywords:** First-episode schizophrenia, Prosody, Social cognition, Emotion recognition, Schizophrenia-spectrum

## Abstract

Studies indicate that people with schizophrenia experience deficits in their ability to accurately detect emotions, both through facial expressions and voice intonation (i.e., prosody), and that functioning and symptoms are associated with these deficits. This study aimed to examine how facial emotion and affective prosody recognition are related to functioning and symptoms in a first-episode schizophrenia sample. Further, in light of research suggesting variable emotion-specific performance in people with schizophrenia, this study explored emotion-specific performance. Participants were 49 people with a recent first episode of schizophrenia taking part in a larger RCT. Results revealed that affective prosody recognition was significantly correlated with both role and social functioning. Regarding associations with psychiatric symptoms, facial emotion recognition was significantly, negatively associated with all three positive symptom scales, whereas affective prosody recognition was significantly, negatively associated with disorganization only. Emotion-specific analyses revealed that for affective prosody, participants were most accurate in recognizing anger and least accurate for disgust. For facial emotion recognition, participants were most accurate in recognizing happiness and least accurate for fear. Taken together, results suggest that affective prosody recognition is important for social and role functioning in people with first-episode schizophrenia. Results also suggest that this group may struggle more to identify negative emotions, though additional work is needed to clarify this pattern in affective prosody and determine real-world impact on social interactions.

## 1 Introduction

Research indicates that people with schizophrenia experience deficits in their abilities to accurately detect emotions compared to healthy controls. In literature examining people with longer-term schizophrenia-spectrum disorders, deficits in facial emotion recognition are well-established (Kohler et al., 2010). Research also suggests that people with schizophrenia experience deficits in their ability to detect affective prosody, or the emotions portrayed through voice intonation (Hoekert et al., 2007), although this literature is not as extensive as that involving facial emotion recognition. Deficits in both facial emotion and affective prosody recognition are evident in individuals at clinical high risk for schizophrenia before a first psychotic episode (Amminger et al., 2011; Corcoran et al., 2015) and help to predict which individuals at clinical high risk go on to develop a psychotic episode (Corcoran et al., 2015). Furthermore, emotion recognition deficits are present to the same degree of severity in prodromal, first episode, and chronic phases of schizophrenia (Green et al., 2011), and the emotional processing deficit after a first episode is relatively stable across five years (McCleery et al., 2016). Taken together, these observations suggest that emotion recognition deficits are core features of schizophrenia-spectrum illnesses that precede psychotic symptoms and likely contribute to their development.

Facial emotion recognition deficits in schizophrenia-spectrum disorders have been found to predict poor functional outcomes, including both community and social functioning (Fett et al., 2011; Irani et al., 2012), and to be related to symptom severity (Ventura et al., 2013). Some research indicates that deficits in affective prosody recognition are also negatively related to functional outcomes (Brekke et al., 2005; Hooker and Park, 2002; Pijnenborg et al., 2009; Vaskinn et al., 2008) and symptoms (Ventura et al., 2013). However, research on affective prosody specifically has mostly been conducted in samples of participants with longer-term illness, with relatively little research available examining relationships with symptoms in earlier phases of the illness (i.e., first-episode schizophrenia). Studies indicate that people with first-episode schizophrenia are impaired in affective prosody recognition as compared to healthy participants (Amminger et al., 2012, Amminger et al., 2011; Caletti et al., 2018; Edwards et al., 2001; Kucharska-Pietura et al., 2005; Thompson et al., 2012). Additionally, some studies suggest that deficits in prosody recognition in first-episode patients are related to increased symptomatology (Caletti et al., 2018; Edwards et al., 2001), but results are inconsistent as to related symptom domains. Furthermore, other studies have found no relationship with symptoms (Amminger et al., 2011; Kucharska-Pietura et al., 2005), and a final study indicates that increased depression positively affects the ability to accurately detect sadness in affective prosody (Herniman et al., 2017). Further research is needed to clarify the nature of the relationship between deficits in prosody recognition and symptomatology in first-episode schizophrenia; additionally, research systematically examining relationships with the symptom clusters of disorganization and reality distortion (Ventura et al., 2010) would clarify our understanding of how prosody recognition is connected to positive symptoms.

Only one study to our knowledge has examined the relationship of affective prosody recognition with functioning in first-episode schizophrenia. Yet, this is an important area for investigation. Emotion recognition, one aspect of the social cognitive processes supporting social interactions more broadly, is integral to functioning in any area requiring social contact – including domains such as school or work (Green et al., 2008). Caletti et al. (2018) found that affective prosody recognition was not correlated with global functioning in first-episode schizophrenia. This study had several strengths, including a large sample. However, functioning was measured using the Global Assessment of Functioning (GAF), which produces a single score and has been criticized for conflating functioning and symptomatology (Burns and Patrick, 2007; Cornblatt et al., 2007). This type of measurement cannot separately assess domains of functioning, such as role functioning (i.e., school/job functioning and independent living skills) and social functioning (i.e., quality and quantity of interpersonal relationships; Cornblatt et al., 2007). Thus, nuanced examination of how functioning is related to affective prosody recognition in first-episode schizophrenia is needed.

While it is important to investigate associations between emotion recognition, functioning, and symptoms, it is also important to fully understand the nature of deficits in affective prosody recognition. While the amygdala has been implicated in emotion recognition generally (Aleman and Kahn, 2005), facial emotion recognition and affective prosody recognition rely on differential sensory processes, which could also suggest differences in how deficits are expressed or related to other illness features. Deficits in auditory processing and pitch perception are present in schizophrenia patients and linked to reduced recognition of affective prosody, but not facial emotion (Jahshan et al., 2013; Leitman et al., 2005); dysfunction in both the primary auditory cortex and the medial prefrontal cortex have also been implicated in deficits in prosody recognition (Lin et al., 2018). Alternatively, abnormal visual scanpaths in schizophrenia have been linked to deficits in facial emotion recognition (Toh et al., 2011).

One particular aspect of affective prosody recognition that is of interest is emotion-specific performance. Numerous studies suggest a relatively clear pattern of emotion-specific performance for facial emotion recognition, as reviewed by Edwards et al. (2002) and Pomarol-Clotet et al. (2010) – people with schizophrenia tend to most accurately identify positive emotions, like happiness and surprise, and least accurately identify fear, specifically, along with other negative emotions. Similar patterns have been identified for facial emotion recognition in first-episode samples (Amminger et al., 2011). Research on affective prosody recognition is less clear. One early study grouped emotions by valence and found that people with schizophrenia performed significantly worse for negative emotions than for positive in a video emotion recognition task that combines facial, prosodic, and other contextual cues (Bell et al., 1997). Another early study of first episode participants grouped facial and prosody tasks together, revealing a specific deficit in the recognition of fear and sadness with the combined scores (Edwards et al., 2001). A more recent study in first episode individuals found that in prosody, participants displayed greater difficulty in identifying anger than other emotions (Amminger et al., 2011), and another found that first episode patients with symptoms of depression were better at identifying sadness than those without depression (Herniman et al., 2017). It has not been the norm to include emotion-specific performance comparisons in the published literature, but this type of analysis may help us to better understand the nature of the deficits in visual versus auditory emotion recognition.

To address gaps in prosody research in those with first-episode schizophrenia, we examined the associations between affective prosody recognition, functioning, and symptoms. Since the field has considerably more knowledge regarding facial emotion recognition than affective prosody recognition in patients with first-episode schizophrenia, we also examined associations with facial emotion recognition in order to determine whether the patterns of correlations between these two sensory modalities for perceiving emotions are similar or different. Based on past research suggesting the importance of facial emotion recognition capabilities for functioning in people with schizophrenia, we expected to find a similar relationship in our first-episode schizophrenia sample for affective prosody recognition, i.e., deficits associated with lower functioning in both role and social domains. We expected this relationship despite the lack of association presented by Caletti et al. (2018) due to our use of more nuanced measurement of functioning. We also expected to find that deficits in affective prosody recognition would be associated with greater experience of reality distortion and disorganization. Lastly, based on a body of research suggesting that performance by people with schizophrenia on emotion recognition tasks varies by specific emotion, we examined emotion-specific performance for both affective prosody and facial emotion recognition tasks. We hypothesized for facial emotion recognition that performance on positive emotions, such as happiness, would be significantly better than for negative emotions, such as fear. Analyses regarding affective prosody recognition were exploratory.

## 2 Method

### 2.1 Participants

Participants included 49 first-episode schizophrenia patients receiving outpatient psychiatric treatment at the UCLA Aftercare Research Program as part of a randomized controlled trial examining the impact of aerobic exercise on cognitive training effects (Nuechterlein et al., 2016) (NIMH R34MH102529). Data used here came from baseline assessments conducted as part of that RCT, prior to randomization. To be eligible, participants had to have a confirmed DSM-IV diagnosis of schizophrenia, schizoaffective disorder, depressed type, or schizophreniform disorder. Participants were not eligible if their psychotic symptoms started more than two years prior to study entry. Additional eligibility criteria included: age between 18 and 45 years; no known neurological disorder; premorbid IQ of 70 or greater; fluent in English and sufficiently acculturated so as to not invalidate research measures; and residence likely to be within commuting distance to UCLA for ongoing services. Notably, participants were not excluded for past substance use disorder unless there was evidence of moderate or severe alcohol or substance use disorder in the 6 months prior to study entry, or if there was evidence that psychotic symptoms were triggered by substance use. At the time of baseline testing, patients at the UCLA Aftercare Research Program are engaged with a clinical case manager and psychiatrist and are considered to be stable enough to engage in the randomized controlled trial interventions.

All participants provided written informed consent for the project, using procedures approved by the UCLA institutional review board. Thirty-four participants were male (69%) and fifteen were female (31%). Participants' average age was 22.4 (SD = 3.9), ranging from 18 to 35. On average, participants had completed 12.8 years of education (SD = 1.4). Fifteen participants were White (31%), thirteen were Black (26%), and twenty-one reported mixed race or “other” (43%). Nineteen (39%) participants reported they were Hispanic.

### 2.2 Measures

#### 2.2.1 Affective prosody recognition

The Prosody Task (Juslin and Laukka, 2001) assesses ability to accurately detect emotions from affective vocal samples by having respondents listen to an audio clip and then choose which of five emotions (or no emotion) is exhibited. The five emotions include happiness, sadness, anger, fear, and disgust. Audio stimuli were developed by Juslin and Laukka (2001) and involve two statements with neutral content (one declarative sentence and one question) said by each of four actors (two female, two male). Actors were instructed to portray each emotion twice for each statement – once at weak emotional intensity and once at strong emotional intensity – resulting in 16 trials for each emotion. Each actor also said each statement once with no emotion (i.e., 8 neutral trials), for a total of 88 trials across emotions. For this study, the total number correct was calculated out of 80, omitting responses for neutral stimuli.

#### 2.2.2 Facial emotion recognition

The Facial Emotion Identification Test (FEIT) (Horan et al., 2009) assesses ability to accurately detect emotions from facial expressions by displaying a picture of a face and having respondents choose which of six emotions (or no emotion) is exhibited. The six emotions include happiness, sadness, anger, fear, surprise, and disgust. Pictorial stimuli come from the Ekman picture set (Ekman, 2003). Each emotion was presented eight times throughout the task, in addition to eight neutral stimuli, for a total of 56 images. For this study, the total number correct was calculated out of 48, omitting responses to neutral stimuli.

#### 2.2.3 Functioning

Functioning was assessed using two interviewer-rated scales. The Global Functioning Scale (GFS) (Cornblatt et al., 2007; Niendam et al., 2006) was developed specifically to evaluate functioning in young adults. The GFS has two subscales, Role and Social. Both are rated on a 10-point scale with well-defined behavioral anchors. The GFS Role subscale evaluates school and job functioning and independent living skills, and the GFS Social subscale evaluates the overall quality and quantity of social interactions.

The Role Functioning Scale (RFS) (Goodman et al., 1993) was also used to assess functioning because of its more nuanced subscales: Independent Living, Working Productivity, Immediate Social Network Relationships, and Family Network Relationships. Notably, the RFS parses social functioning into functioning in friendships (Immediate Social Network Relationships) and functioning in family relationships (Family Network Relationships). Each subscale of the RFS is rated on a 7-point scale.

#### 2.2.4 Symptoms

A 24-item version of the Brief Psychiatric Rating Scale (BPRS) (Lukoff et al., 1986; Ventura et al., 1993) was used to assess the severity of positive and negative symptoms. Positive symptoms were further broken down into Reality Distortion and Disorganization domains (Ventura et al., 2013). Negative symptoms were also assessed with the Scale for the Assessment of Negative Symptoms (SANS) (Andreasen, 1984). Only a SANS total score was used for the purposes of this study, and the Attention item was omitted from scoring due to its low association with the other SANS items.

### 2.3 Procedure

Participants were recruited from local healthcare facilities in Los Angeles. Diagnostic eligibility for enrollment was determined via interview by trained raters (Ventura et al., 1998) with the Structured Clinical Interview for DSM-IV Axis I Disorders – Patient Edition (SCID-I/P) (First et al., 2001) and supplemental informant information prior to any research testing. Upon enrollment at the UCLA Aftercare Research Program, participants began receiving clinical services with a case manager and psychiatrist immediately. All participants were prescribed second-generation antipsychotic medication. Participants typically underwent baseline testing 2–4 months after clinic entry, depending on clinical need and stabilization. Participants were administered the testing battery by trained research assistants. Participants were compensated $25/h for research testing.

### 2.4 Analyses

To examine hypothesis 1, that prosody and facial emotion recognition would be positively associated with functioning and negatively associated with symptoms, we calculated Pearson's correlations. Correlations were considered significant at *p* *≤* .05. To examine hypothesis 2, that participants' performance would vary for specific emotions within prosody and facial emotion recognition tasks, we conducted one-way within-subjects analyses of variance (ANOVA) for each task, using Bonferroni correction to adjust for multiple pairwise comparisons (with a resultant *p*-value of 0.005 for prosody and 0.003 for facial emotion recognition). Lastly, we conducted exploratory, unadjusted correlations to determine the extent to which emotion-specific performance was related between sensory modalities. All analyses were conducted in SPSS version 24.

## 3 Results

Mean scores on measures of symptoms and functioning can be found in Table 1. Results of correlational analyses examining associations between prosody and facial recognition total scores, functioning, and symptoms can be found in Table 2. Regarding functioning, the Prosody Task was significantly associated with GFS Role Functioning (i.e. work and school performance) and RFS Social Functioning (i.e., functioning in social relationships with friends). The FEIT was not significantly associated with any functioning measure, though the analysis examining RFS Social Functioning approached statistical significance (*p* = .07). Neither task had significant associations with GFS Social Functioning or RFS Work Functioning or Independent Living. Regarding symptoms, the Prosody Task was significantly, negatively associated with BPRS Disorganization only. The FEIT, on the other hand, was significantly, negatively correlated with all three positive symptom scales on the BPRS (Disorganization, Reality Distortion, and Positive Symptoms), suggesting positive symptoms more broadly were related to performance on the FEIT. Neither affective recognition task was associated with negative symptoms measured by BPRS or SANS.

**Table 1:** – Mean scores on symptom and functioning measures.

Domain	Mean (SD)
BPRS Reality Distortion	2.39 (1.58)
BPRS Disorganization	1.45 (0.50)
BPRS Positive Symptoms	2.24 (1.19)
BPRS Negative Symptoms	2.80 (1.45)
SANS Total	2.30 (1.21)
Role Functioning (GFS)	3.98 (2.34)
Social Functioning (GFS)	5.22 (1.77)
Work Functioning (RFS)	3.11 (1.66)
Independent Living (RFS)	3.41 (1.13)
Family Functioning (RFS)	4.73 (1.04)
Social Functioning (RFS)	3.55 (1.53)

Note. GFS = Global Functioning Scale (range 1–10); RFS = Role Functioning Scale (range 1–7); BPRS = Brief Psychiatric Rating Scale (range 1–7); SANS = Scale for the Assessment of Negative Symptoms (range 0–5).

**Table 2:** – Correlations of prosody and facial affect recognition with functioning and symptom severity.

	Prosody total	Facial affect total
Role Functioning (GFS)	0.32⁎	0.19
Social Functioning (GFS)	0.17	0.21
Work Functioning (RFS)	0.18	0.04
Independent Living (RFS)	0.18	0.16
Family Functioning (RFS)	0.23	0.12
Social Functioning (RFS)	0.38⁎	0.28
BPRS Reality Distortion	−0.21	−0.28⁎
BPRS Disorganization	−0.31⁎	**−**0.50⁎⁎
BPRS Positive Symptoms	−0.28	−0.37⁎⁎
BPRS Negative Symptoms	0.01	−0.02
SANS total	−0.19	−0.24

*Note*. GFS = Global Functioning Scale; RFS = Role Functioning Scale; BPRS = Brief Psychiatric Rating Scale; SANS = Scale for the Assessment of Negative Symptoms.

⁎ Correlation is significant at p < .05 (2-tailed).

⁎⁎ Correlation is significant at *p* < .01 (2-tailed).

Participants' overall scores on each task as well as their scores on specific emotions are summarized in Table 3. Results of within-subject ANOVA revealed significant differences between specific emotions for both the Prosody Task [*F*(3.29, 157.73) = 22.94, *p* < .001] and the FEIT [*F*(3.80, 182.51) = 25.99, *p* < .001]. For the Prosody Task, participants had the highest scores for anger and sadness, both of which were significantly higher than scores for disgust, happiness, and fear. Participants scored most poorly for disgust, which was significantly lower than scores for all other emotions. For the FEIT, consistent with hypotheses, participants had the highest scores for happiness, which was significantly higher than all other emotions, followed by surprise, which was significantly higher than disgust, fear, and sadness, though not anger. Participants scored most poorly for fear, which was significantly lower than scores for all other emotions. Detailed pairwise comparison tables are available in the supplemental material.

**Table 3:** – Task and emotion mean scores.

Task/emotion	Mean (SD)	Average % correct
Affective prosody recognition total	28.3 (7.9)	35.4%
Happiness	5.4 (2.8)	33.8%
Anger	7.3 (2.2)	45.6%
Disgust	3.5 (2.3)	21.9%
Sadness	7.0 (2.8)	43.8%
Fear	5.2 (2.6)	32.5%
Facial emotion recognition total	38.2 (5.9)	79.6%
Happiness	7.8 (1.0)	97.5%
Surprise	7.2 (1.4)	90.0%
Anger	6.8 (1.5)	85.0%
Disgust	5.9 (2.2)	73.8%
Sadness	6.2 (2.0)	77.5%
Fear	4.4 (2.3)	55.0%

*Note*. Higher scores on both tasks indicate better performance. For the Prosody Task, the total possible score was out of 80 and each emotion score was out of 16. For the Facial Emotion Identification Test, the total possible score was out of 48 and each emotion score was out of 8.

Regarding correlations for emotion-specific performance between sensory modalities (i.e., auditory vs. visual), results suggested performance between the tasks was largely orthogonal for happiness (*r* = 0.03, *p* = .848), anger (*r* = 0.18, *p* = .207), disgust (*r* = 0.07, *p* = .628), and sadness (*r* = 0.08, *p* = .590). Accuracy of identification for fear was significantly correlated between the two tasks (*r* = 0.34, *p* = .017).

## 4 Discussion

Our results indicate that affective prosody recognition is related to both role and social functioning, while facial emotion recognition only exhibited a trend-level association (*p* = .07) with social functioning. These findings are consistent with the view that emotion recognition deficits could be contributing to deficits in the ability of people with first-episode schizophrenia to adequately function in the real world, both in relationships with friends and in normative young adult roles. Unlike the one other study to investigate prosody recognition with functioning in early psychosis (Caletti et al., 2018), we found correlations with multiple domains of functioning on different measures (role functioning on GFS, social functioning on RFS). These associations with functioning were most likely uncovered due to more nuanced measurement, allowing functioning in varied domains to be parsed. Of interest, affective prosody recognition was associated with role functioning on the GFS, but not with work functioning or independent living on the RFS. The GFS Role scale was specifically designed to measure a range of roles that are typical of adolescents and young adults, including school/work and everyday living tasks, so is may be more sensitive in this age range than the adult-oriented RFS (Cornblatt et al., 2007). This increased specificity and relevance to young people may have allowed the association with role functioning to emerge, while less tailored scales (such as the RFS) may miss more nuanced aspects of role functioning during this age range.

While the GFS was more able to detect associations with role functioning in our sample, neither affective prosody nor facial emotion recognition were significantly associated with social functioning on the GFS. On the other hand, the RFS Immediate Social Network Functioning scale, which measures social functioning outside of the family, detected relationships with both types of emotion recognition (albeit at a trend level for facial emotion recognition). Neither prosody nor facial affect recognition was associated with social functioning within the family network on the RFS, so the separation of these two social functioning domains may be critical. This suggests both affective prosody and facial emotion recognition may be more important in friendships than in family relationships. Perhaps family relationships are more dependent on the family's commitment to being helpful to the patient after a psychotic episode rather than the patient's ability to accurately recognize family member's emotional reactions. Additional research, perhaps qualitative in nature, would be helpful in determining the driving factors in family relationships that negate the influence of deficits in emotion recognition.

Regarding symptoms, affective prosody recognition was associated only with symptoms of disorganization, while facial emotion recognition was associated with both disorganization and reality distortion. This suggests that positive symptoms might be more strongly influenced by the ability to detect emotion in faces than to interpret emotion in vocal intonation. That both types of emotion recognition are associated with disorganization in first-episode schizophrenia patients is consistent with meta-analyses that have suggested that disorganization in schizophrenia is associated with both affective prosody and facial emotion recognition, with larger effect sizes than for either reality distortion or negative symptoms (Ventura et al., 2013). Indeed, our results follow meta-analytic findings of Ventura et al. (2013) closely – facial emotion recognition was significantly associated with both reality distortion and disorganization, while affective prosody recognition was only associated with disorganization.

Why might reality distortion be associated with facial emotion recognition but not affective prosody recognition? As others have hypothesized, reality distortion could possibly lead to increased misattributions regarding facial expressions (Ventura et al., 2013), which could, theoretically, then work to reinforce delusional ideology. However, consistent with literature demonstrating early onset of social cognitive deficits prior even to development of symptoms, certain sensory processes that lead to deficits in facial emotion recognition may also influence development or severity of symptoms. One potential factor is abnormalities in visual scan paths found in people with schizophrenia, indicating that these individuals spend less time observing salient features of the face and thus, draw conclusions regarding emotion expression based on a paucity of information (Toh et al., 2011). While this finding has not yet been replicated in first-episode schizophrenia to our knowledge, research indicates that the pattern holds even in participants from the general population who report subthreshold psychotic experiences (Hillmann et al., 2015). In this context, our findings align with cognitive conceptualizations of delusion formation suggesting that paranoid content develops when a person makes judgments based on limited information (Bell et al., 2006; Freeman, 2016) – i.e., assuming our participants have visual scanpath tendencies similar to those found in the literature (biasing visual attention away from salient emotional features), their determination of facial emotion expression based on limited relevant information may be linked to heightened experience of paranoid of persecutory delusions. Reality distortion in our sample often reflects paranoid symptomatology and may therefore be associated with visual scanning patterns that do not focus on salient aspects of emotional faces. The same processes would not be relevant for affective prosody recognition.

Lastly, our findings for emotion-specific performance in first-episode schizophrenia indicate that participants' pattern of performance was not the same between facial and prosody modalities. This was supported by within-subjects analyses, suggesting participants were better at identifying different emotions in each condition and that performance on the same emotion across conditions were largely unrelated (with the exception of fear). This could suggest that different emotions present cues that are legitimately less clear in some aspects of expression than others (e.g., vocal representation of disgust less easily discerned than facial representation), though a healthy comparison sample would be needed to verify this possibility. If true, social cognitive interventions may want to target specific emotions in emotion recognition activities. Alternatively, visual abnormalities (Green et al., 2003; Phillips et al., 2000) or auditory processing deficits (Leitman et al., 2005) in schizophrenia may play a larger role in determining relative accuracy across different emotions.

While these results add to the literature regarding emotion-specific performance for affective prosody recognition, in the real world, people with first-episode schizophrenia likely interpret facial and prosodic cues within their full social context. Additional studies are needed with contextually rich stimuli, such as the Bell-Lysaker Emotion Recognition Task (BLERT) (Bell et al., 1997), to inform our understanding of how people with first-episode schizophrenia interpret multiple sources of information in tandem to identify a given emotion.

Our study has limitations. Results should be replicated in a sample with healthy control participants for comparison; the within-sample comparisons made in this study cannot inform whether participants with first-episode schizophrenia displayed a deficit in performance relative to a healthy sample. Further, surprise was included only in the facial emotion recognition task (FEIT). Future studies should attempt to incorporate surprise as an emotion in prosody tasks, as well. Our sample was also relatively small. It is possible that true associations that are small in magnitude were missed due to limited statistical power. Relatedly, we chose not to correct for alpha inflation in correlational analyses, due to the early nature of our research; future work should examine these relationships in larger samples to determine whether they replicate. Moreover, due to the cross-sectional nature of this data, we cannot determine causal relationships, nor how these constructs may relate to each other over time. Lastly, this study cannot inform the relative importance of emotion recognition for prediction of functional outcomes as compared to non-social elements of cognition. Future work should investigate the relative importance of emotion recognition as a predictor of functional outcomes and examine the impact of non-social elements that may contribute to deficits in emotion recognition, such as cognitive control and sensory deficits (Dondaine et al., 2014).

Taken together, our results suggest that recognition of affective prosody is important for social and role functioning in people with first-episode schizophrenia. This finding has clinical implications. Interventions designed to target social cognitive skills (e.g., Combs et al., 2007; Horan et al., 2009) may improve social functioning via improvement of emotion recognition skills. Our study highlights that affective prosody recognition, an element not often targeted in social cognitive interventions, may play an important role in how emotion recognition impacts functioning. Lastly, our results suggest that people with first-episode schizophrenia may struggle more to identify negative emotions, though additional work is needed to clarify this pattern in affective prosody and determine the impact this has on real-world, contextually-rich social interactions.

## Declaration of Competing Interest

Keith Nuechterlein, Ph.D., received supplemental support from Janssen Scientific Affairs for a medication component of the larger project, has received unrelated research grants from Genentech and Posit Science, and has been a consultant to Astellas, Genentech, Janssen, Medincell, Otsuka, Takeda, and Teva. Joseph Ventura, Ph.D., has received funding from Brain Plasticity, Inc., Genentech, Inc., and Janssen Scientific Affairs, LLC, and has served as a consultant to Boehringer-Ingelheim, GmbH, and Brain Plasticity, Inc. Kenneth Subotnik, Ph.D., has received funding from Janssen Scientific Affairs, LLC through grants to Dr. Nuechterlein, has served as a consultant to Alkermes, Inc, and Medincell, Inc, and has been on the speaker's bureaus for Janssen Canada and Otsuka America Pharmaceutical, Inc. Kelsey Bonfils, Ph.D., declares no conflicts of interest.

## Appendix A Supplementary data

Supplementary tables Image 1
