# Supplementary material for: Affective prosody and facial emotion recognition in first-episode schizophrenia: Associations with functioning & symptoms
Source: Schizophr Res Cogn. 2019 May 25;18:100153. doi: 10.1016/j.scog.2019.100153 (PMC6718049; doi:10.1016/j.scog.2019.100153)
Supplement: Supplementary file 1 — Supplementary tables [file mmc1.docx]

Supplementary Material, “Affective prosody and facial emotion recognition in first-episode schizophrenia: Associations with functioning & symptoms”

Table 1: Detailed pairwise comparison tables for within-subject ANOVA of affective prosody recognition

| (I) emotion | (J) emotion | Mean Difference (I-J) | Std. Error | Sig. | 95% Confidence Interval for Difference | |
| --- | --- | --- | --- | --- | --- | --- |
|  |  |  |  |  | Lower Bound | Upper Bound |
| 1 afraid | 2 | -2.041^*^ | 0.34 | 0.000 | -3.05 | -1.03 |
|  | 3 | 1.714^*^ | 0.47 | 0.006 | 0.33 | 3.10 |
|  | 4 | -0.12 | 0.52 | 1.000 | -1.66 | 1.42 |
|  | 5 | -1.755^*^ | 0.55 | 0.024 | -3.36 | -0.15 |
| 2 angry | 1 | 2.041^*^ | 0.34 | 0.000 | 1.03 | 3.05 |
|  | 3 | 3.755^*^ | 0.41 | 0.000 | 2.56 | 4.95 |
|  | 4 | 1.918^*^ | 0.44 | 0.001 | 0.63 | 3.21 |
|  | 5 | 0.29 | 0.44 | 1.000 | -1.02 | 1.59 |
| 3 disgusted | 1 | -1.714^*^ | 0.47 | 0.006 | -3.10 | -0.33 |
|  | 2 | -3.755^*^ | 0.41 | 0.000 | -4.95 | -2.56 |
|  | 4 | -1.837^*^ | 0.40 | 0.000 | -3.03 | -0.65 |
|  | 5 | -3.469^*^ | 0.41 | 0.000 | -4.68 | -2.26 |
| 4 happy | 1 | 0.12 | 0.52 | 1.000 | -1.42 | 1.66 |
|  | 2 | -1.918^*^ | 0.44 | 0.001 | -3.21 | -0.63 |
|  | 3 | 1.837^*^ | 0.40 | 0.000 | 0.65 | 3.03 |
|  | 5 | -1.633^*^ | 0.46 | 0.010 | -3.00 | -0.27 |
| 5 sad | 1 | 1.755^*^ | 0.55 | 0.024 | 0.15 | 3.36 |
|  | 2 | -0.29 | 0.44 | 1.000 | -1.59 | 1.02 |
|  | 3 | 3.469^*^ | 0.41 | 0.000 | 2.26 | 4.68 |
|  | 4 | 1.633^*^ | 0.46 | 0.010 | 0.27 | 3.00 |

Note. Bonferroni adjustment was utilized to determine significance in pairwise comparison, using SPSS procedures. In this method, significance values are multiplied by the number of comparisons, then assessed using the typical value of p ≤ .05. Significance values presented here are adjusted; i.e., they have been multiplied by 10 (for an effective p-value of .005). If, when multiplied, the p-value reaches or exceeds 1, it is denoted as a 1 in the table. Table 2: Detailed pairwise comparison tables for within-subject ANOVA of facial emotionrecognition

| Measure: Ekman | | | | | | |
| --- | --- | --- | --- | --- | --- | --- |
| (I) emotion | (J) emotion | Mean Difference (I-J) | Std. Error | Sig. | 95% Confidence Interval for Difference | |
|  |  |  |  |  | Lower Bound | Upper Bound |
| 1 anger | 2 | 0.837 | 0.36 | 0.365 | -0.28 | 1.95 |
|  | 3 | 2.388^*^ | 0.36 | 0.000 | 1.26 | 3.51 |
|  | 4 | -1.020^*^ | 0.23 | 0.001 | -1.73 | -0.31 |
|  | 5 | 0.592 | 0.35 | 1.000 | -0.49 | 1.67 |
|  | 6 | -0.49 | 0.23 | 0.529 | -1.19 | 0.21 |
| 2 disgust | 1 | -0.837 | 0.36 | 0.365 | -1.95 | 0.28 |
|  | 3 | 1.551^*^ | 0.39 | 0.004 | 0.35 | 2.76 |
|  | 4 | -1.857^*^ | 0.35 | 0.000 | -2.92 | -0.79 |
|  | 5 | -0.245 | 0.41 | 1.000 | -1.51 | 1.02 |
|  | 6 | -1.327^*^ | 0.36 | 0.008 | -2.43 | -0.22 |
| 3 fear | 1 | -2.388^*^ | 0.36 | 0.000 | -3.51 | -1.26 |
|  | 2 | -1.551^*^ | 0.39 | 0.004 | -2.76 | -0.35 |
|  | 4 | -3.408^*^ | 0.33 | 0.000 | -4.41 | -2.41 |
|  | 5 | -1.796^*^ | 0.38 | 0.000 | -2.96 | -0.64 |
|  | 6 | -2.878^*^ | 0.36 | 0.000 | -4.00 | -1.76 |
| 4 happy | 1 | 1.020^*^ | 0.23 | 0.001 | 0.31 | 1.73 |
|  | 2 | 1.857^*^ | 0.35 | 0.000 | 0.79 | 2.92 |
|  | 3 | 3.408^*^ | 0.33 | 0.000 | 2.41 | 4.41 |
|  | 5 | 1.612^*^ | 0.30 | 0.000 | 0.69 | 2.54 |
|  | 6 | .531^*^ | 0.14 | 0.005 | 0.11 | 0.95 |
| 5 sad | 1 | -0.592 | 0.35 | 1.000 | -1.67 | 0.49 |
|  | 2 | 0.245 | 0.41 | 1.000 | -1.02 | 1.51 |
|  | 3 | 1.796^*^ | 0.38 | 0.000 | 0.64 | 2.96 |
|  | 4 | -1.612^*^ | 0.30 | 0.000 | -2.54 | -0.69 |
|  | 6 | -1.082^*^ | 0.33 | 0.026 | -2.09 | -0.08 |
| 6 surprised | 1 | 0.49 | 0.23 | 0.529 | -0.21 | 1.19 |
|  | 2 | 1.327^*^ | 0.36 | 0.008 | 0.22 | 2.43 |
|  | 3 | 2.878^*^ | 0.36 | 0.000 | 1.76 | 4.00 |
|  | 4 | -.531^*^ | 0.14 | 0.005 | -0.95 | -0.11 |
|  | 5 | 1.082^*^ | 0.33 | 0.026 | 0.08 | 2.09 |

Note. Bonferroni adjustment was utilized to determine significance in pairwise comparison, using SPSS procedures. In this method, significance values are multiplied by the number of comparisons, then assessed using the typical value of p ≤ .05. Significance values presented here are adjusted; i.e., they have been multiplied by 15 (for an effective p-value of .003). If, when multiplied, the p-value reaches or exceeds 1, it is denoted as a 1 in the table.
